# The Relationship Between Social Media Use and Well-Being: The Mediating Role of Social Support

**DOI:** 10.1093/geroni/igab046.1648

**Published:** 2021-12-17

**Authors:** Xin Yao Lin, Margie Lachman

**Affiliations:** 1 Brandeis University, Waltham, Massachusetts, United States; 2 Brandeis University, Brandeis University, Massachusetts, United States

## Abstract

Frequent social media usage can have negative effects on well-being, but the mechanisms involved are unclear. This study explored the mediating role of giving and receiving support. Using the Midlife in the United States Refresher eight-day daily diary study (N=782, age 25-75), multilevel structural equation modeling examined the hypothesized relationships at both the within- (intraindividual) and between-person (interindividual) levels. Results showed that at the within-person level, days with more social media use were associated with a larger proportion of time giving support and worse well-being (less positive affect and more stress, negative affect, and loneliness). At the between-person level, more social media use was associated with worse well-being. Giving support, but not receiving support, mediated the relationship between social media use and well-being at the within, but not the between-person level. Discussion focuses on ways to address the negative consequences of social media use related to social connections and well-being.

